# Measurement of glucose metabolism in the occipital lobe and frontal cortex after oral administration of [1-13C]glucose at 9.4 T

**DOI:** 10.1177/0271678X221104540

**Published:** 2022-05-27

**Authors:** Theresia Ziegs, Johanna Dorst, Loreen Ruhm, Nikolai Avdievitch, Anke Henning

**Affiliations:** 1 High‐Field MR Center, Max Planck Institute for Biological Cybernetics, Tübingen, Germany; 2 IMPRS for Cognitive and Systems Neuroscience, Tübingen, Germany; 3 Advanced Imaging Research Center, University of Texas Southwestern Medical Center, Dallas, Texas, USA

**Keywords:** Glucose metabolism, glutamatergic metabolism, human brain, proton magnetic resonance spectroscopy, ultra-high field strengths

## Abstract

For the first time, labeling effects after oral intake of [1-13C]glucose are observed in the human brain with pure 1H detection at 9.4 T. Spectral time series were acquired using a short-TE 1H MRS MC-semiLASER (Metabolite Cycling semi Localization by Adiabatic SElective Refocusing) sequence in two voxels of 5.4 mL in the frontal cortex and the occipital lobe. High-quality time-courses of [4-13C]glutamate, [4-13C]glutamine, [3-13C]glutamate + glutamine, [2-13C] glutamate+glutamine and [3-13C]aspartate for individual volunteers and additionally, group-averaged time-courses of labeled and non-labeled brain glucose could be obtained. Using a one-compartment model, mean metabolic rates were calculated for each voxel position: The mean rate of the TCA-cycle (Vtca) value was determined to be 1.36 and 0.93 μmol min^−1^ g^−1^, the mean rate of glutamine synthesis (Vgln) was calculated to be 0.23 and 0.45 μmol min^−1^ g^−1^, the mean exchange rate between cytosolic amino acids and mitochondrial Krebs cycle intermediates (Vx) rate was found to be 0.57 and 1.21 μmol min^−1^ g^−1^ for the occipital lobe and the frontal cortex, respectively. These values were in agreement with previously reported data. Altogether, it can be shown that this most simple technique combining oral administration of [1-13C]Glc with pure 1H MRS acquisition is suitable to measure metabolic rates.

## Introduction

Human brain metabolism can be studied noninvasively using Carbon-13 (13C) MR spectroscopy after the administration of 13C labeled substrates, e.g. glucose (Glc). By consuming the 13C labeled Glc, its 13C nuclei are transferred to pyruvate/lactate by the glycolysis and subsequently further downstream to tricarboxylic acid cycle (TCA) intermediates, which convert e.g. to glutamate (Glu) and glutamine (Gln). In the past, the spectral changes induced by the 13C label incorporation were mostly observed via two different approaches: direct 13C MRS detection^1–4^ or indirect 1H-[13C] MRS methods^4–7^ using editing techniques such as POCE.^8–11^ On the basis of the large number of publications on methods and applications in this field, the reader is referred to respective review articles,^12–17^ which are explaining and discussing i.e. the details of these methods and the choice of different labeled substrates.

Instead of using direct or indirect 13C methods to measure metabolic rates, a new technique was invented by Boumezbeur et al. showing the feasibility of using conventional 1H MRS without a 13C channel.^18^ With this method, the incorporation of 13C nuclei is detectable due to heteronuclear scalar coupling of 1H and 13C: When 13C nuclei are incorporated into metabolites, the respective 1H MRS signals of 12C-bonded protons decrease and the signals from 13C-bonded protons increase. Using conventional 1H MRS, several technical challenges of direct and indirect 13C MRS can be avoided as there is no need for special 13C hardware, like broadband amplifiers, multinuclear transmitters, radiofrequency (RF) coils as well as no special 13C sequences are required. In addition, problems of high specific absorption rates in brain tissue associated with heteronuclear decoupling sequences are not an issue for conventional 1H MRS.^18^ These advantages make the 1H MRS approach attractive for those MR centers without the possibility of 13C hardware and scanner software as is the case on most clinical MR systems. In contrast, conventional 1H MRS is usable in most MR centers and benefits from broad expertise concerning RF coils and sequences, which enable the application of an optimal set-up for the study aims.

So far, there are only a few other studies using conventional 1H MRS to measure 13C label incorporation into downstream metabolites in animals.^19,20^ The first applications to humans have been presented only recently. In 2015 An et al.,^21^ in 2017 Bartnik-Olson et al.^22^ and in 2020 Dehghani et al.^23^ applied Boumezbeur’s technique in humans. However, they show data from very few metabolites only: An et al. presented spectral data from different time-points only with decreasing [4-12C]Glu and [4-12C]Gln resonance signals but no uptake curves; Bartnik-Olson et al. showed temporal Glu signal decrease of patients with epilepsy and controls; and Dehghani et al. showed uptake curves with the percent enrichment for [4-13C]Glu, [4-13C]Glx and [3-13C]Glx.

In the present study, we follow the incorporation of the 13C nuclei into [4-13C]Glu, [4-13C]Gln, [3-13C]Glx, [2-13C]Glx, and [3-13C]Asp after oral administration of [1-13C]Glc for the individual subjects and could additionally achieve the time course of the labeled and unlabeled Glc in the brain averaged across subjects.

In addition, we show the possibility to measure the rate of glutamine synthesis Vgln, the rate of the TCA-cycle Vtca and the exchange rate between cytosolic amino acids and mitochondrial Krebs cycle intermediates Vx for the group means, which no human study using Boumezbeur’s technique did so far.

## Methods

### Human subjects

In this study, labeling effects after the oral administration of [1-13C]Glc were measured in two different brain regions on 11 healthy volunteers (5 female, 6 male, mean age 29 ± 2 years). The measurements were done on 9 of the volunteers in a voxel in the frontal cortex and on 7 of them in a voxel placed in the occipital lobe; so, 5 volunteers were scanned twice with 3–13 weeks between the two sessions. Before the measurement started, the volunteers gave their written informed consent according to the local research ethics regulations, the current version of the Declaration of Helsinki, DIN EN ISO 14 155 and were approved by the Institutional Review Board of the University of Tübingen.

### [1-13C]Glc administration

The volunteers fastened for 9 hours overnight before the measurement started. Before and after the scan the blood sugar level was tested with a glucometer (Accu-Check, Roche Diabetes Care GmbH, Mannheim, Germany) to detect possible hypoglycemia after the Glc administration. Hypoglycemia was not encountered for any subject. For each volunteer, a solution containing 0.75 g of [1-13C]Glc (Aldrich Chemical Company, Miamisburg, Ohio, USA; API for clinical studies) per kilogram body weight was prepared and the subjects drank the Glc solution after the acquisition of the first spectrum with 64 averages (see below for the measurement details).

### Data acquisition

All measurements were performed using a 9.4 T whole-body MR scanner (Magnetom, Siemens Healthineers, Erlangen, Germany). A home-built 4-channel surface coil was used for the measurement in the occipital lobe^24^ as it is described in Dorst et al.^25^ and an 8-channel Tx/16-channel Rx volume coil in 3-loop surface transmit mode was used for the voxel in the frontal cortex.^26^ More details about the coil setup can be found in the Supplementary Material and Figure S1.

Sagittal and transversal gradient-echo scout images were acquired for the positioning of the voxel (15 × 18 × 20 mm^3^ = 5.4 ml) either in the frontal cortex or the occipital lobe; see Figure 1 for the voxel position and corresponding sample spectra. The voxel in the frontal cortex was placed in the gray matter region centered on the interhemispheric fissure. And the occipital voxel was placed in the right hemisphere 0.5–1 cm from the interhemispheric fissure touching the occipital horn of the lateral ventricle in the right upper corner keeping in mind that the voxel should not be too close to the skull to avoid lipid contamination due to chemical shift displacement error. First-order and second-order B_0_ shimming were performed using FAST(EST)MAP,^27^ and then a voxel-based power calibration was executed.^28,29^ The spectra were measured using a short TE 1H MC-semiLASER sequence; details can be found in Giapitzakis et al.^30^ with optimizations of the spoiling, phase cycling and crushers later described by Dorst et al.^25^ (TE/TR = 24/5000 ms, 64 averages (Ave), acquisition time = 512 ms, bandwidth = 8000 Hz, sequence duration = 5.67 min). A water-reference signal (Ave = 16) as well as a macromolecular spectrum^31,32^ (TR = 8 s, Ave = 32) were taken from the same voxel. After the baseline 1H MRS measurement block was executed, the scanner table was pulled out and the volunteers drank the Glc solution as fast as possible (if possible lying flat while drinking with a straw). After the oral intake of [1-13C]Glc, a localizer was applied to verify the position of the voxel. In case of a changed head position the same voxel positioning and calibration procedure were applied as described above including gradient-echo scout sequences to relocate the voxel, B_0_ shimming and power calibration before starting the acquisition of a series of MC-semiLASER 1H MRS blocks with 64 averages. The time of repositioning and calibration took 14–21 minutes. If no repositioning of the voxel was needed, MC-semiLASER blocks of 64 averages were applied immediately after the oral Glc intake. In these cases, the time between Glc intake and start of the data acquisition was 5–9 minutes. In each of the volunteers as many as possible MC-semiLASER 1H MRS blocks of 64 averages were acquired to fill a maximum possible scan time of 2 hours.

**Figure:**
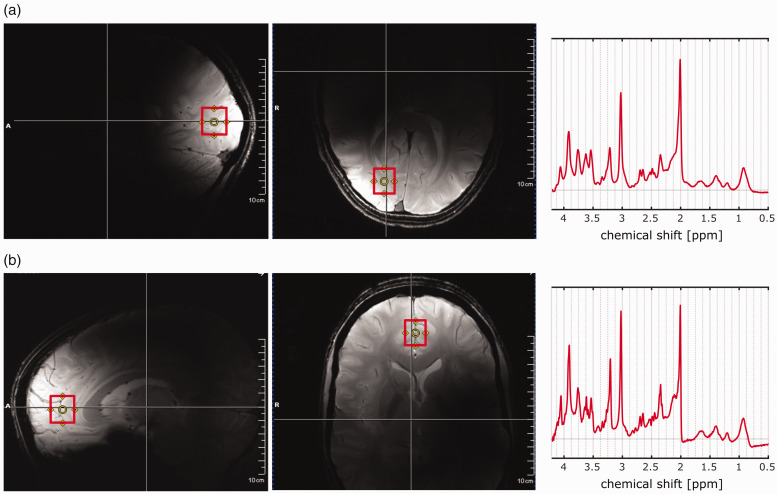
Figure 1. Position of the voxel (15 × 18 × 20 mm^2^) in the occipital lobe (a) and the frontal cortex (b) along with the corresponding sample spectra.

### Data processing

Raw MRS data were processed with in-house-written code in Matlab (version 2016a; MathWorks, Natick, MA) as described in detail before^25,30,31,33^ and here summarized: (1) replace the data points after 250 ms with zeros; (2) frequency and phase alignment; (3) MC subtraction for the metabolite spectra and the macromolecular data; (4) averaging; (5) eddy current correction using the MC water signal; (6) combine coils using a singular value decomposition method; (7) peak alignment to the NAA signal or the water signal for the water data; (8) removing residual water signal using HSVD for the spectral data; and (9) truncation at 150 ms with subsequent zero filling.

The calculation of the difference spectra needed for the calculation of the metabolite enrichments is explained in the next subsection since extra steps after fitting the pre-Glc-intake spectrum were needed.

### Spectral fitting

The 1H MRS data were fitted with LCModel (V6.3‐1L)^34^ with spectral basis sets simulated with VeSPA (version 0.9.5^35^) using full quantum mechanical density matrix calculations for the semi-LASER sequence.^36^ Three separate basis set were generated: for the pre-Glc-intake, for the post-Glc-intake spectra and for the Glc changes in the downfield region.

#### Pre-Glc-intake basis set

The first basis set was used to analyze the baseline spectra acquired before the oral [1-13C]Glc intake. The basis set consists of the mean macromolecular spectrum of the volunteers from the occipital lobe (due to lower variability and noise of the data in the occipital lobe than the frontal cortex^37^) and 13 metabolites: ascorbic acid (Asc), aspartate (Asp), creatine (Cr), γ-aminobutyric acid (GABA), Glc, Gln, Glu, glutathione (GSH), lactate (Lac), myo-inositol (mI), NAA, NAAG, phosphocreatine (PCr), phosphorylethanolamine, scyllo-inositol (scyllo), taurine (Tau), and total choline (tCho, glycerophosphocholine (GPC) + phosphocholine (PCho)). J-coupling constants and chemical shifts were taken from Govindaraju et al.^38,39^ except for the J-coupling constants for GABA, which were taken from Near et al.^40^ The spectra were fitted between 0.6 ppm and 4.2 ppm including water scaling and the LCModel parameter dkntmn (minimum spacing of the spline baseline knots in ppm, cannot exceed one third of the fitted range,^41^ which reflects the spline baseline stiffness) was set to 0.25.

#### Post-Glc-intake basis set

**Figure 2.**
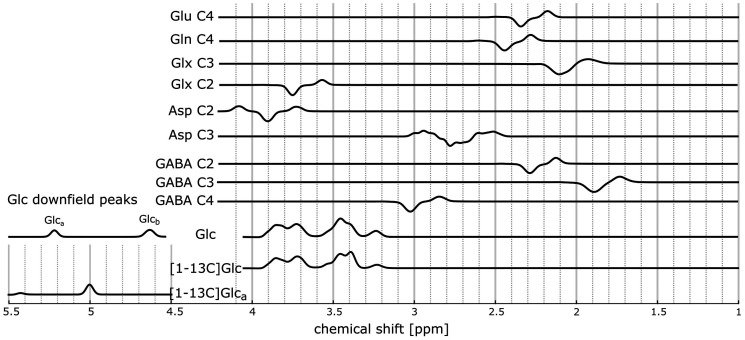
Simulated LCModel basis spectra for metabolites, which are 13C labeled at different carbon positions for the LCModel fit of post-Glc-administration: Glutamate and glutamine labeled at position C4 (Glu C4, Gln C4, respectively), the mix of glutamate and glutamine labeled at position C3 and C2 (Glx C3, Glx C2, respectively), aspartate labeled at position C2 and C3 (Asp C2, Asp C3, respectively) as well as GABA labeled at position C2, C3 and C4 (GABA C2, GABA C3, GABA C4, respectively). These basis spectra were simulated using the method of Boumezbeur et al. as described in the method section to account for the decrease for 12C-bonded protons and the increase of 13C-bonded protons at the same time. In the bottom three rows the unlabeled Glc spectrum for the upfield and the downfield area, the labeled Glc spectrum for the upfield area and the [1-13C]Glc_α_ were seen for the downfield area. The Glc and the [1-13C]Glc basis sets are indistinguishable for LCModel. Thus, only the unlabeled Glc basis set was used in the LCModel fits.

For fitting the changes caused by the incorporation of the 13C nuclei into downstream metabolites, difference spectra were used. No linewidth adjustments were applied. Before subtracting the pre-Glc administration baseline spectrum from the post-Glc administration spectra to obtain a time series of difference spectra, two changes were made: First, the fitted tCr CH_3_ peak at ∼3.0 ppm was subtracted from the pre-Glc spectrum to obtain a pre-Glc spectrum without the tCr CH_3_ peak. Thus, the tCr CH_3_ will not be subtracted when calculating the difference spectra so that the difference spectra still contained its tCr CH_3_ peak, which ensured better LCModel fitting stability since LCModel takes this Cr peak as reference for its first fit iteration. Secondly, the same procedure was used with the upfield Glc peaks: The fitted Glc peaks were subtracted from the pre-Glc spectrum before calculating the difference spectra. The idea behind the second adjustment is the following: the complex multiplets of Glc are often very poorly fitted due to the low concentration and the broad range of chemical shifts between 3.1 ppm–4 ppm. However, the Glc quantification can benefit from the increasing Glc level due to the Glc intake. To keep this advantage, the total Glc level should remain in the difference spectra. Since the unlabeled Glc peaks are indistinguishable for LCModel from the labeled Glc in the upfield region (as seen in Figure 2), the basis set for the post-Glc-intake difference spectra contained the unlabeled Glc metabolite peaks of alpha- and beta-Glc (ratio of 0.36 to 0.64) in addition to the tCr CH_3_ peak at ∼3.0 ppm. Furthermore, the LCModel basis set for the post-Glc intake difference spectra contained metabolite peaks of Glu, Gln, Asp, Lac, and GABA, which were expected to change after the 13C incorporation with the heteronuclear J-coupling constants taken from de Graaf.^42^ Which metabolites are labeled by the incorporation of the 13C nuclei after [1-13C]Glc intake is illustrated in previous publications.^12,43,44^ To account for the advantage to simultaneously detect the singal of 12C-bonded and 13C-bonded protons, a method described in Boumezbeur et al.^18^ was used, who fitted the difference spectra and combined the corresponding 13C uptake and 12C decrease in one basis set. The method shall be described shortly through the example of Glu labeled at the C4 position:

- If the C4 position of Glu is labeled, the 13C nucleus couples to the 4H-Glu protons at 2.34 and 2.35 ppm with 126.7 Hz. Glu spin systems with and without 13C at the C4 position were simulated with VeSPA. Both simulations were subsequently subtracted to account for the decrease of the unlabeled C4 peak and the increase of the labeled C4 peaks. The basis spectrum was called Glu C4.
- This was done for Gln labeled at the position C4 (Gln C4), for the mix of Glu and Gln labeled at C3 and C2 (Glx C3 and Glx C2, respectively), for GABA labeled at C2, C3 and C4 (GABA C2, GABA C3 and GABA C4), Lac labeled at C3 (Lac C3) and Asp labeled at C2 and C3 (Asp C2 and Asp C3). The simulated basis spectra are shown in Figure 2. Since Lac changes could not be seen in any volunteer, the Lac C3 spectrum was not included in Figure 2. Glu and Gln at the C3 and C2 positions were combined to Glx since the chemical shifts significantly overlap and cannot be distinguished at 9.4 T.

Additionally, the Cho CH_3_ peak at ∼3.2 ppm and the NAA CH_3_ peak at 2.0 ppm were added to the basis set to account for subtraction errors, which are mainly caused by the repositioning of the volunteer after the Glc intake. The spectra were fitted between 0.6 ppm and 4.2 ppm including water scaling and the LCModel parameter dkntmn was set to 999, which is a stiff baseline.

#### Post-Glc-intake Glc enrichment basis set

Since the fit of the post-Glc-intake difference spectra (previous paragraph) provided only information of the total Glc (Glc_tot_) concentration, an alternative had to be found to obtain the time-courses for the labeled Glc. So, a closer look has to be taken at the downfield region: While the [1-12C]Glc_β_ peak at 4.630 ppm is overlaid by the water resonance, the decrease in the [1-12C]Glc_α_ peak at 5.216 ppm is generally detectable. Unfortunately, the increase of the corresponding [1-13C]Glc_α_ peaks at 5 and 5.43 ppm could not be reliably detected since the first peak is too close to the water signal, and the second peak is overlaid by an artifact, see Figure 2 for the simulated Glc downfield basis set and Figure S2 for an example of the artifact. Therefore, the post-Glc-intake spectra were fitted with an increased ppm range from 5.1 to 6.2 ppm. In addition, to all metabolites used in the pre-Glc fit, the [1-12C]Glc_α_ peak at 5.216 ppm and all corresponding 13C labeled metabolites were included in the basis set. Only the fitted values for the [1-12C]Glc_α_ peak were taken into account for further calculations. The resulting time course for the [1-12C]Glc_α_ concentration was too variable for single volunteers and thus, the concentrations were averaged across subjects for each position and subsequently fitted with an exponentially decaying function. From the mean time course of Glc_tot_ obtained from the difference spectra and exponential fit of the time course of [1-12C]Glc_α_ from the downfield fits, the time course of the [1-13C]Glc can be calculated as follows: Since Glc_tot_ is the sum of [1-13C]Glc and [1-12C]Glc, and the level of [1-12C]Glc can be calculated by [1-12C]Glc = [1-12C]Glc_α_/0.36, the following equation is obtained and used for the calculation of the [1-13C] Glc enrichment: [1-13C]Glc = Glc_tot_–[1-12C]Glc_α_/0.36.

### Quantification

Percent Enrichments (PE) of labeled Glu, Gln and Asp were calculated by dividing the concentration of the labeled metabolite of the post-Glc-intake spectra by the concentration of the non-labeled metabolite of the pre-Glc-intake spectrum for each volunteer separately. For metabolic rate calculations, the PEs were averaged across subjects for both voxel positions. The percent enrichment of the [1-13C]Glc was determined from the time courses of Glc_tot_ from the upfield difference spectra and the time course obtained by the exponential fit of the downfield [1-12C]Glc_α_ peak changes at ∼5.2 ppm with the following equation: [1-13C]Glc/Glc_tot_ = 1 - [1-13C]Glc_α_/0.36/Glc_tot_.

### Metabolic rate determination

The metabolic rates were calculated using the Matlab-based program CWAVE (Version 3.6) from Graeme F. Mason.^45^ This program provides a graphic interface to describe the mass and isotopic flows from the labeled substrate for an arbitrary metabolic model. The program thus solves the differential equations numerically with a 4th/5th order Runge-Kutta^10^ algorithm, calculates corresponding metabolic rates of the model and statistical distributions of uncertainties using Monte-Carlo analysis. In this study, a simple one-compartment model was used describing the incorporation of 13C nuclei from the 1st carbon position of Glc into Glu and Gln at the 4th carbon position and subsequently into Glu and Gln at the 3rd carbon position. The time course of [1-13C]Glc is used as the input function, see previous publications^12,43,44^ for illustrations of the labeling pathways. The model (adapted from^2,46,47^) and the corresponding differential equations can be found in Supplementary Figure S3 and Supplementary Table T1. The one-compartment model was used with the fewest assumptions possible to avoid overfitting and to have a robust estimate of the resulting rates. The present results were compared to the previous literature. Thus, the rate of the TCA-cycle Vtca, glutamine synthesis Vgln and the exchange rate between cytosolic amino acids and mitochondrial Krebs cycle intermediates Vx could be determined for the mean time courses for both voxel positions.

## Results

**Figure 3.**
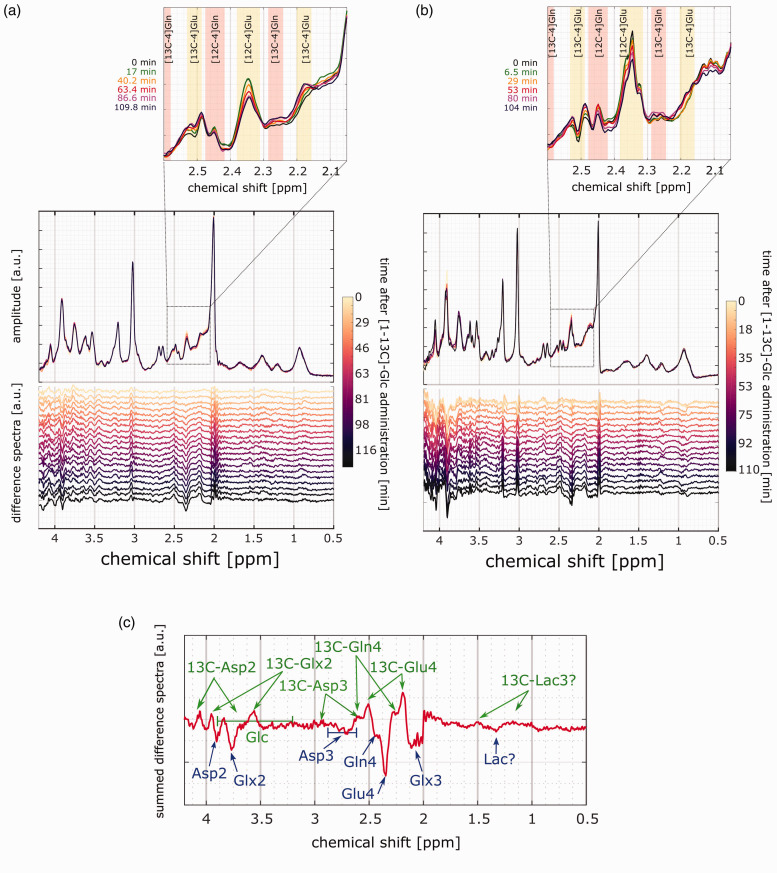
Time series of spectra and difference spectra from (a) occipital lobe and (b) frontal cortex. The changes for the 12C-bonded H4-glutamate and -glutamine signals are highlighted in the zoomed figure above for a few selected time points.and (c) Sum of the last difference spectrum for all volunteers. Decreasing metabolite peaks with 12C-bonded protons are marked in blue and increasing satilite peaks due to the coupling to 13C are marked in green. In the lower panel, subtraction errors at the NAA ^2^CH_3_, Cr CH_3_ and Cho CH_3_ peaks are additionally shown.

Time series of spectra from two volunteers from the occipital lobe and the frontal cortex are shown in Figure 3(a) and (b). Most dominant is the labeling effect in the region between 2 and 2.5 ppm: the [4-12C]Glu peak at ∼2.34 ppm decreases and the [4-13C]Glu peaks at 2.18 and 2.5 ppm increase. The same is seen for the [4-12C]Gln peak at ∼2.44 ppm and the corresponding [4-13C]Gln satellite peaks. The zoomed part of Figure 3 makes these spectral pattern changes better visible by a selection of spectra from six time points with color coding of frequency ranges corresponding to the Glu and Gln peaks in yellow and red, respectively.

The summation of all volunteers' difference spectra between the respective first and last acquired spectra for both voxel positions in Figure 3(c)) reveals changes due to the labeling at the 4th Glu and Gln carbon position, the 3rd Glx and Asp carbon position and the 2nd Glx and Asp carbon position. The changes of [3-13C]Lac were minor. Metabolite peaks with increasing amplitude are marked in green and those with decreasing amplitude are indicated in blue. Additionally, subtraction errors are seen at the tCr CH_3_ peak at 3.0 ppm, the tCho CH_3_ peak at ∼3.2 ppm and the NAA CH_3_ peak at 2.0 ppm. The region from 3.8 to 4.2 ppm shows artifacts and thus cannot be interpreted.

**Figure 4.**
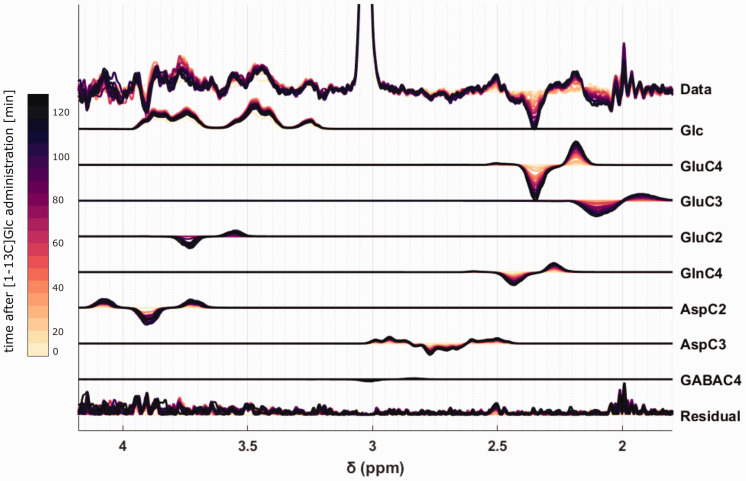
Difference spectra and fitted metabolite basis sets for the metabolites of interests with the remaining Cr CH_3_ peak at 3 ppm. In addition, the residual of the difference spectra and the fit are displayed. Sample data from one volunteer from the occipital lobe. Colors indicate time points from light to dark (yellow for short after the Glc intake to black at the very end of the measurement).

The difference spectra, the fitted metabolite spectra and the fit residual for different time points are shown for one sample volunteer in Figure 4; colors indicate different time points. The increase of the (negative) 12C peaks and the (positive) 13C satellite peaks are clearly detectable.

In general, the data quality is better in the occipital lobe than the frontal cortex in terms of SNR and variability between time points, due to the high receive SNR surface coil specifically made for SVS in the occipital lobe and higher impact of motion in the frontal cortex. See Supplementary Material and Supplementary Figure S4 and S5 for more details about FWHM and SNR of the measurements in both voxel positions.

**Figure 5.**
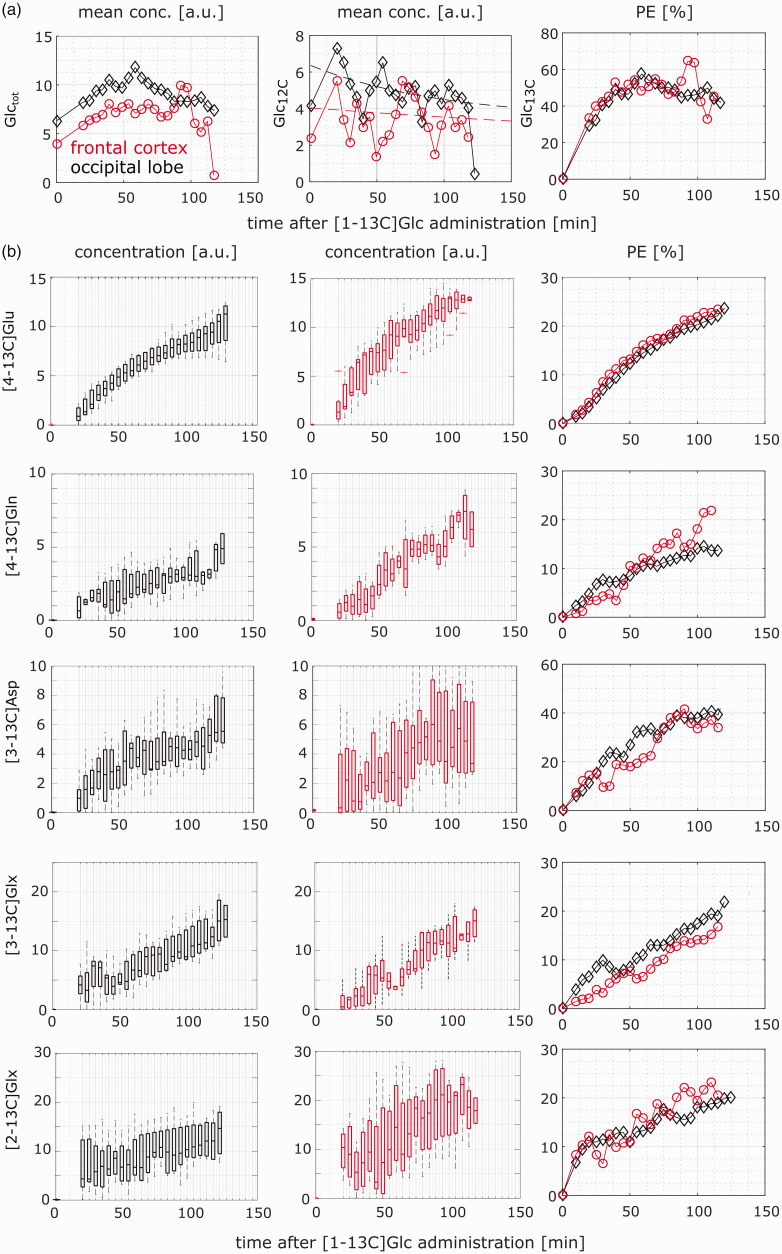
(a) The first row shows mean concentrations across all volunteers in the occipital lobe (black) and frontal cortex (red). The columns show data Glctot, Glc12C in addition to an exponential fit and Glc13C and In (b) the time courses for different labeled metabolies is shown. The range of concentration for each volunteer for the occipital lobe (first column), frontal cortex (second column) and the percent enrichment (PE) calculated from the mean concentration for each position is presented. For the calculation of the mean concentration as well as the percent enrichment, data from volunteers, which are too far from the median, were removed. See methods section for more information.

Mean time courses for the total, labeled and unlabeled Glc are shown in the first row of Figure 5(a)). The data from the single volunteers are summarized in boxplots in Supplementary Figure S6. The Cramér-Rao-Lower-Bound (CRLB) for the fitted Glc_tot_, calculated from LCModel, was 4 ± 2% and 11 ± 6% (mean ± std across subjects) for the measurements of the occipital lobe and the frontal cortex, respectively. The time courses of [4-13C]Glu, [4-13C]Gln, [3-13C]Asp, [3-13C]Glx, [2-13C]Glx for each volunteer are summarized in boxplots for the occipital lobe (black, 1st column) and the frontal cortex (red, 2nd column), see 2nd row and the following of Figure 5. The third column of Figure 5 presents the percent enrichment (PE) calculated from the mean concentration for both voxel positions. The maximum enrichment of the brain Glc was 58.5% and 54.8% for the occipital lobe and frontal cortex, respectively. For the other metabolites, the following enrichments were achieved after 2 h of measurement: [4-13C]Glu: 24% and 24%, [4-13C]Gln: 15% and 20%, [3-13C]Asp: 41% and 37%, [3-13C]Glx: 19% and 17%; [2-13C]Glx: 19% and 23% for the occipital lobe and frontal cortex, respectively.

For the calculation of the mean concentration as well as the PE, those data were removed, where the fit of the metabolite is zero for more than half of the time points or show clearly incorrect time courses, which strongly differ from the median.

**Figure 6.**
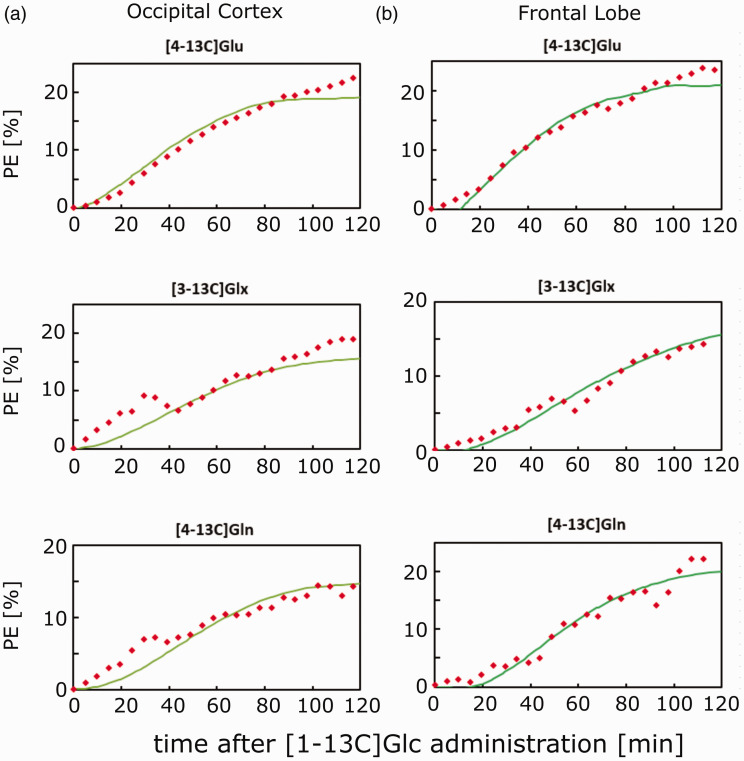
Experimental mean percent enrichment for [4-13C]Glu, [3-13C]Glx and [4-13C]Gln for both voxel positions (red dots) and the fitted curve from CWave (green) with a single-compartment model in (a) for the occipital lobe and in and (b) for the frontal cortex.

The rates Vgln, Vtca and Vx were calculated for both measured positions and are shown in Table 1 including the results of the Monte-Carlo error analysis from CWave. In Figure 6, the respective data and fitted curves from CWave are seen for [4-13C]Glu, [4-13C]Gln and [3-13C]Glx.

**Table 1.** Calculated rates with a 1-compartment metabolic model using CWave (mean, std): the combined rate of glutamine synthetase and glutaminase Vgln, TCA cycle rate Vtca and the TCA cycle intermediates exchange rate Vx.

	Vgln (μmol min^–1^ g^–1^)	Vtca (μmol min^–1^ g^–1^)	Vx (μmol min^–1^ g^–1^)
OCC	0.23 ± 0.03	1.36 ± 0.05	0.57 ± 0.05
FRONT	0.45 ± 0.03	0.93 ± 0.04	1.21 ± 0.09

## Discussion

In this study, effects of 13C labeling in 1H MR spectra from two voxel positions in healthy human brain after oral intake of [1-13C]Glc were presented acquired with an MC-semiLASER sequence at 9.4 T. Using a pure 1H MRS technique without the presence of any 13C hardware, as introduced by Boumezbeur et al.,^18^ uptake curves for a larger number of metabolites could be obtained than before with this approach: temporal data from labeled and unlabeled Glc, [4-13C]Glu, [4-13C]Gln, [3-13C]Asp, [3-13C]Glx and [2-13C]Glx are shown herein. The data quality of the averaged time course across subjects was sufficient to obtain rate estimates for the TCA cycle rate Vtca, the glutamine synthesis rate Vgln and the exchange rate of TCA cycle intermediates with cytosolic amino acids Vx, which are in agreement with literature values. None of the previous human studies using a pure 1H MRS technique calculated these rates and only two animal studies presented data for Vtca.^18,20^

### Oral administration

So far, the infusion of 13C labeled substrates was common to investigate brain metabolism and only a few studies used an oral administration: One used an oral intake exclusively^48^ and three other studies compared infusion and oral protocols.^1,4,49^ Both administrations have certain advantages and disadvantages, which will be discussed below:

1. One downside of the oral intake is the delayed labeling of the metabolites due to the gastrointestinal Glc absorption, which is avoided by intranvenous (i.v.) administration. The delay leads to prolonged examination times^1,4^ and thus potentially in increasing motion artifacts and scan time costs.
2. Secondly, a larger percentage of Glc retained in liver^50^ results in a higher amount of Glc needed in an oral protocol than in i.v. administration to reach similar enrichment levels. Moreno et al.^49^ showed that comparable results are achieved in a low dose infusion protocol with only 1/3 of the Glc amount of an oral dose. On the other hand, the costs per gram 13C labeled Glc approved for i.v. administration might be significantly higher than the costs for Glc applicable for oral intake due to the need for a higher clinical trial grade and tests from a specialized pharmacy guaranteeing the safety of the i.v. administration. Furthermore, with an oral intake the effort of the study set-up and the associated costs for additional material (i.v. pumps, i.v. lines, Teflon needles etc.) and people (phlebotomist/doctor/nurse) are largely reduced.
3. While oral and i.v. administration lead to similar mean rates of Vtca and Vgln, Mason et al.^4^ objects that the oral intake leads to a several-fold bigger standard deviation (SD). In contrast to the i.v. set-up, where the bolus infusion leads to a quick Glc PE increase, the oral intake results in a delayed Glc enrichment, see point 1 above, and thus, the uptake curve of Glc PE resemble the time courses of the consequently labeled metabolites more. As a consequence, the simulated curves become less sensitive to the rate constants,^4^ which requires higher precision and accuracy of these time courses than in an i.v. protocol. Nonetheless, Mason et al. worked out that the major part of the uncertainty in an oral set-up in comparison to an i.v. administration is caused by imprecise knowledge about the kinetics of the glucose arrival in the brain. As most studies determine the brain Glc PE from the blood Glc PE, the limiting factors are: uncertainties of sampling time (length of the i.v. line) and differences between enrichment of the arterial blood Glc in the brain and venous plasma Glc sampling location.^4^ This reason could partly be solved by measuring the Glc enrichment directly in vivo in the brain, which is generally possible as Pfeuffer et al.^51^ showed in rat brain at 9.4 T using a 1H-[13C]MRS. Unfortunately, the individual subject's Glc enrichment could not be obtained in the present study but only group means are reported. To focus on the detection of downfield Glc peaks in humans could be a valid aim for future studies to access individual brain Glc PEs.
4. Beside these downsides, the major advantages of oral administration are the reduced effort in the set-up and the significantly higher comfort for the volunteers. This enlarges the pool of potential volunteers and patients to participate in neuroscientific and clinical trials enormously, since many more people are willing to drink a sugar solution, but may refuse a catheterization e.g. in case of needle phobia.

### Time courses of labeled metabolites

Most previous studies showing uptake curves in human brain focused on the detection of [4-13C]Glu, [4-13C]Gln, [3-13C]Glu, [3-13C]Gln, or combination of Glu and Gln.^3,4,10,22,23,46,52–54^ Although some studies could also detect the labeling of additional metabolites in their MR spectra, only a very few studies displayed uptake curves of other metabolites: [2-13C]NAA,^43^ [2-13C]Glu,^2,55^ [2-13C]Gln,^2,55^ [3-13C]Lac,^1,56^ [2,3-13C]Asp,^1^ and [2-13C]Asp.^43,55,56^

In this study, time courses of a relatively broad range of metabolites could be obtained at once: 13C labeled and 12C unlabeled Glc, [4-13C]Glu, [4-13C]Gln, [3-13C]Asp, [3-13C]Glx, [2-13C]Glx. The time courses of labeled Lac, labeled GABA and [2-13C]Asp could not be achieved. Lactate did not seem to change and the concentration of GABA is too low to detect respective changes.

#### Glc enrichment

In many cases, the LCModel fit of Glc_tot_ in the upfield region in 1H single voxel spectra even in ultra-high field MRS measurements is incorrect or even unsuccessful.^29,57^ But the Glc_tot_ data of this human brain 9.4 T study is of high quality. However, the fit of the [1-12C]Glc_α_ peak in the downfield region exhibits a high variance, which is why an exponential fit on the mean [1-12C]Glc_α_ time course across subjects was used to derive the [1-13C]Glc PE.

The shape of the time course of [1-13C]Glc is similar in both voxel positions and resembles the time courses from other oral Glc intake studies.^49,58^ The enrichments in the current study are higher than the value of 38.3% Moreno et al.^49^ reported, but they used 0.65 g/kg body weight, which is 0.1 g/kg body weight Glc less in their oral protocol. Unfortunately, many studies show only the plasma Glc enrichment instead of the brain Glc enrichment. Assuming that the fractional enrichment of plasma and brain Glc is similar,^9,20,49^ both values can be compared: The 13C MRS study from Mason et al.^4^ using the same [1-13C]Glc amount in the oral set-up, showed plasma Glc PE of more than 75%. Most infusion studies report peak values of the brain Glc enrichment of about 60–70% (in animals^20,59^ or humans^49,60^) which depends on the amount of Glc infused, but the amount of Glc of the maintenance dose after the initial bolus is not reported in most studies. Altogether, the results of this study are in line with previous reports.

#### Glutamate and glutamine enrichment

The [4-13C]Glu enrichment curves for both voxel positions show good quality time courses with low noise. The concentration of [4-13C]Glu increases faster in the frontal cortex, but both PE curves are fairly the same. The shape of the concentration curves look similar to those reported in previous oral 13C MRS studies:^4,49^ fast increase in the first hour, reduced increase afterwards and Mason et al.^4^ shows that it reaches a saturation plateau after about 3 hours of measurement, which could not be performed in the present study since scan time was restricted to a maximum of 2 hours. A relocation after a break in between as Mason et al. suggested, would add significant uncertainty.

Mason et al.^4^ reported about 20% [4-13C]Glu enrichment for the oral administration with the same Glc amount as used in the present study and the peak enrichment reported by Moreno et al.^49^ was 16% (as already mentioned, they used less Glc). The only study using Boumezbeur’s technique on humans showing enrichment curves was Dehghani et al.^23^ using an infusion protocol leading to approx. 15% PE. Other infusion studies using [1-13C]Glc in humans measured PEs between approx. 17–32%.^2,3,10,46,55^ Many other studies reported the signal intensity in arbitrary units or the concentration in mmol/kg or mM and no fractional enrichment. It can be concluded, that the herein measured maximum PE for [4-13C]Glu is in the range of previously reported values.

The [4-13C]Gln enrichment values are in line with previous results, although the data from the frontal cortex show high variability, and the final enrichment should be treated with caution: The only oral study presenting [4-13C]Gln concentration curves from Mason et al. indicates a PE of approx. 14–17%.^4^ Previous infusion studies reported a PE of approx. 25%.^2,46^

There are only a few literature values from human studies and no data from oral experiments to compare the [3-13C]Glx data: Dehghani et al.^23^ shows an increase of 5–10% after 1 h using Boumezbeur’s technique; and other infusion studies determined a labeling of 23% of [3-13C]Glu after 3 h of measurement.^3,46^ These values are comparable to those measured in the present study.

The PE of [2-13C]Glx is also similar to the previously reported values of 10-15% for [2-13C]Glu and [2-13C]Gln^2^ after 1 h of measurement in an infusion study.

#### Aspartate enrichment

For the 1H MRS data reported herein, only [3-13C]Asp time courses could be detected. Changes in the [2-13C]Asp peaks could not be successfully quantified due to two reasons: First, the spectral range of the [2-12C]Asp peak at 3.9 ppm is disturbed by an artifact, and secondly, one of the satellites peaks due to the 13C labeling occurs at 4.1 ppm, which is strongly influenced, by residual water signal and corresponding baseline errors. The artifact at 3.9 ppm could also cause the high PE values of [3-13C]Asp, if the fitting of Asp in the pre-Glc spectra was underestimated as a consequence. The reference PE values in previous literature are definitely lower than the present values. Two human studies determined PEs of [2-13C]Asp of 12%^43^ and [3-13C]Asp of 27%.^3^

### Metabolic rate calculations

The [4-13C]Glu curves show the best fit for time points <100 min. The uptake curves for time points >100 min are still rising while the fit is not, which indicates that the input function (labeled Glc) is not correctly determined for >100 min. Other reasons for discrepancies of fitted and experimental time courses can be the incompleteness of the one-compartment model. It does not take the regional/cellular heterogeneity^47^ and the compartmentalization of the metabolites, into account e.g. Glu is higher concentrated in neurons and Gln in glia cells. Consequently, the Vtca rate calculated reflects mostly neuronal metabolism.^61^ Since overly sophisticated models come with the risk of overfitting,^62,63^ a more complex analysis with two or even more compartments would have been sensible when individual Glc time-courses would have been accessible in an appropriate quality and additional information could have been obtained such as segmentation of tissue types^10^ or alternative labeling schemes. In addition, it would be crucial to evaluate the influence of different assumptions within the model (concentration of TCA intermediates, dilution rates, etc.) on the fitted rate values (as it was partly done in^47,64^) as well as its robustness. However, such an analysis is beyond the scope of the herein presented study.

Nevertheless, the metabolic rates are in agreement with previous literature: The glutamine synthesis rate Vgln is in the range of the values from oral^4^ and infusion studies.^2,4,46,52,64^ Although Vgln for the frontal cortex is more on the upper limit of the range of rates previously reported. The rate of the TCA cycle in the frontal cortex is similar to previous reports in infusion studies,^2,10,46,52,65^ while the Vtca rate from the occipital lobe is somewhat higher than the highest previously reported values.^10,65^ The rate of the exchange of TCA cycle intermediates with cytosolic amino acids Vx in the occipital lobe was found to be the same as in an infusion study^64^ (and animal studies show similar values for Vx^5,18,47,66^) but the Vx rate for the frontal cortex was about twice as high. Previous studies predict a Vx rate, which is comparable to Vtca,^64,67^ but there seems to be still no consensus of the correct Vx value.^16,68^

The SDs of the metabolic rates in Table 1 are rather small although the SNR of the difference spectra for the labeled metabolites is relatively low, see Supplementary Figure S5. The reasons for this apparent discrepancy are the following: CWave’s calculation of the SDs depends on the variance of the uptake curves. Their variances are relatively small since they result from averaged individual curves and averaging obviously reduces the variance. Particularly, the important time course of [4-13C]Glu is very smooth, see Figure 5. The second explanation is that the fitting procedure takes full advantage of the present method to simultaneously detect the 12C-and the 13C-bonded proton signals by combining both in one basis spectrum (see subsection Post-Glc-intake basis set). Thus, the uptake curves are more accurate than the SNRs from the difference spectra may indicate, since the SNRs were calculated from the maximal peak change of the metabolites only.

### Problems and improvements

Further improvements of the oral study setup can be achieved with the use of [1,6-13C]Glc or [U-13C]Glc, which doubles the sensitivity since two pyruvate molecules can be labeled instead of only one pyruvate molecule from [1-13C]Glc. On the other hand, [1,6-13C]Glc costs about 5 times more than [1-13C]Glc (which was already approx. 5000 euro/person) and is thus not affordable for human studies. [U-13C]Glc would be a less expensive alternative. It would label additional positions, which should have negligible effects on the 1H spectra.^15,42,69^ For the present study, [U-13C]Glc would have been still more expensive than [1-13C]Glc (approx. twice the price of [1-13C]Glc).

A problem of this study was the break within the scan session to drink the labeled Glc. In most cases, the scanner table was pulled out of the whole-body scanner and the volunteers drank the solution with a straw while lying flat; in a few cases, the volunteers had to come out of the head coil completely to drink the solution sitting. Although no volunteer reported problems with choking in the lying position, the possibility of choking has to be kept in mind. The re-positioning of the post-Glc voxel to the original voxel was not in all cases perfect. Although the voxel position was corrected manually by visual inspection, a small possible mismatch of pre-Glc to post-Glc voxel position remains. A possible solution would be to use automatic voxel positioning as it exists at many clinical scanners, but not for the 9.4 T scanner used in this study.

While time courses of many differently labeled metabolites could be determined for most volunteers individually, the Glc time-course is less accurate and could only be evaluated as a mean of all volunteers for each voxel position. Especially, the time-course of the downfield Glc showed high variance, which would be reduced if the artifact at 5.4 ppm could be avoided, and thus, the increasing [1-13C]Glc_α_ peak could be evaluated directly. Additionally, the time courses from both labeled Asp positions could potentially be determined without this artifact. Alternatively, individual brain Glc enrichments could be achieved if blood is drawn (which was not possible in the present study inside a non-clinical setting) to obtain individual rate measurements.

A minor uncertainty is caused by the time the volunteers needed for drinking the Glc solution, so the starting time might vary for about one minute.

## Conclusion

Oral administration of [1-13C]Glc leads to detectable changes in 1H MRS spectra acquired in the human brain at 9.4 T in the occipital lobe as well as in the frontal cortex. From time courses of labeled and unlabeled Glc, [4-13C]Glu, [4-13C]Gln, [3-13C]Glx, [2-13C]Glx and [3-13C]Asp, the corresponding rates Vtca, Vgln and Vx could be calculated for the average across subjects and are in accordance to literature values. These results support the applicability of oral administration of 13C labeled Glc and conventional 1H MRS at ultra-high field to assess metabolic turnover rates in the human brain.

## Supplemental Material

sj-pdf-1-jcb-10.1177_0271678X221104540 - Supplemental material for Measurement of glucose metabolism in the occipital lobe and frontal cortex after oral administration of [1-13C]glucose at 9.4 T Click here for additional data file. Supplemental material, sj-pdf-1-jcb-10.1177_0271678X221104540 for Measurement of glucose metabolism in the occipital lobe and frontal cortex after oral administration of [1-13C]glucose at 9.4 T by Theresia Ziegs, Johanna Dorst, Loreen Ruhm, Nikolai Avdievitch and Anke Henning in Journal of Cerebral Blood Flow & Metabolism

## Data Availability

Data sets, code, and additional information will be provided by the authors upon request.
