# Supplementary material for: Measurement of glucose metabolism in the occipital lobe and frontal cortex after oral administration of [1-13C]glucose at 9.4 T
Source: J Cereb Blood Flow Metab. 2022 May 27;42(10):1890–904. doi: 10.1177/0271678X221104540 (PMC9536126; doi:10.1177/0271678X221104540)
Supplement: sj-pdf-1-jcb-10.1177_0271678X221104540 - Supplemental material for Measurement of glucose metabolism in the occipital lobe and frontal cortex after oral administration of [1-13C]glucose at 9.4 T [file sj-pdf-1-jcb-10.1177_0271678X221104540.pdf]

Supplementary Material

Radiofrequency (RF) Coil Set-up

<u>OCCIPITAL LOBE</u>: The measurement of the spectra in the occipital lobe with a home-built 4Tx/8Rx half-volume coil[1] was performed with the same coil and set-up as described in Dorst et al.[2]. A picture of the coil can be found in Fig. S1A.

<u>FRONTAL CORTEX</u>: The measurement in the frontal cortex were performed with an 8Tx/16Rx volume coil[3] in 3-loop surface transmit mode. The 3-loop surface mode for a different location in the brain is defined in Giapitzakis et al.[4] A picture of the coil set up with the three transmit channels (loops 4, 5, 6) marked is shown in Figure S1B. Figure S1C shows the B1+ field profiles of the 3-channel-mode with 90° phase shift between adjacent loops in comparison to the circular polarized (CP) 8-channel mode(45° phase shift between adjacent loops). As seen in Figure S1C, the B1+ value in the frontal cortex is significantly higher in the 3-channel mode than in the CP mode. Consequently, the local SAR values are higher in the 3-channel-mode too as it can be seen in Figure S1D with maximum local SAR values of 0.66 W/kg for the CP mode and 2.1 W/kg for the 3-channel-mode for an input power of 1W (RMS) at the coil. To account for the increased SAR values for the in vivo measurements, the scanner internal k-factor was changed to the worst case k-factor [1/kg]. The k-factor is used by the scanner to control the global and local SAR. Electromagnetic simulations were done using CST Studio Suite (CST, Darmstadt, Germany) using the virtual multi-tissue model "Duke", seen in Figure S1E.

The unbalanced three-way Wilkinson power splitter used for both coil set-ups is described in Giapitzakis et al. (Supporting Fig. S1C)[4] and Dorst et al. (under revision, Supporting Fig. S1A).

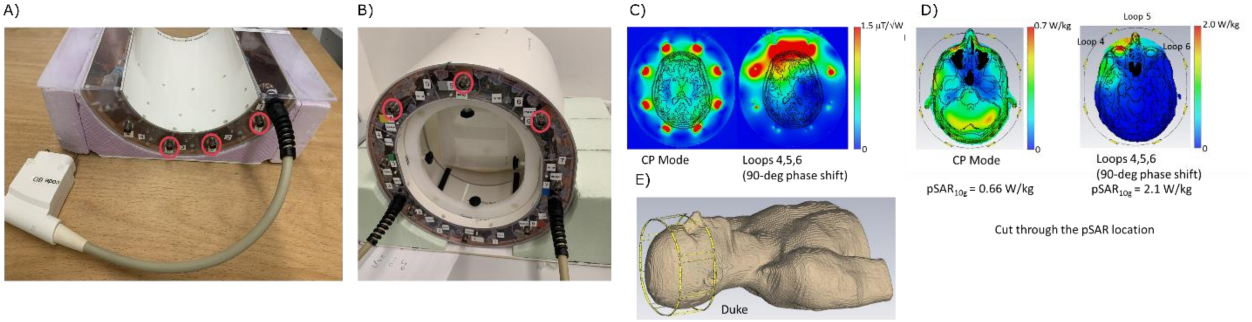

Figure S1: A) Picture of the 4Tx/8Rx half-volume coil used in 3-channel-mode. Transmit channels are marked in red. B) Picture of the 8Tx/16Rx volume coil used in 3-channel mode. Transmit channels are marked in red. C) B1+ field profiles of the 8-channel CP mode and the 3-channel mode. D) Local SAR (averages over 10g of tissue) maps for the CP mode and the 3-channel mode with maximum SAR values of 0.66 W/kg and 2.1 W/kg for the CP mode and the 3-channel mode, respectively. SAR was calculated for 1W of stimulated power at the coil input. Consider the different color scale boundaries for visual reasons. E) The simulation was made for the virtual multi-tissue model "Duke".

Receive Chain Artifact

This artifact occurs symmetrically to the water signal, but is not phase stable. It is potentially caused by phase jittering due to the scanner hardware.

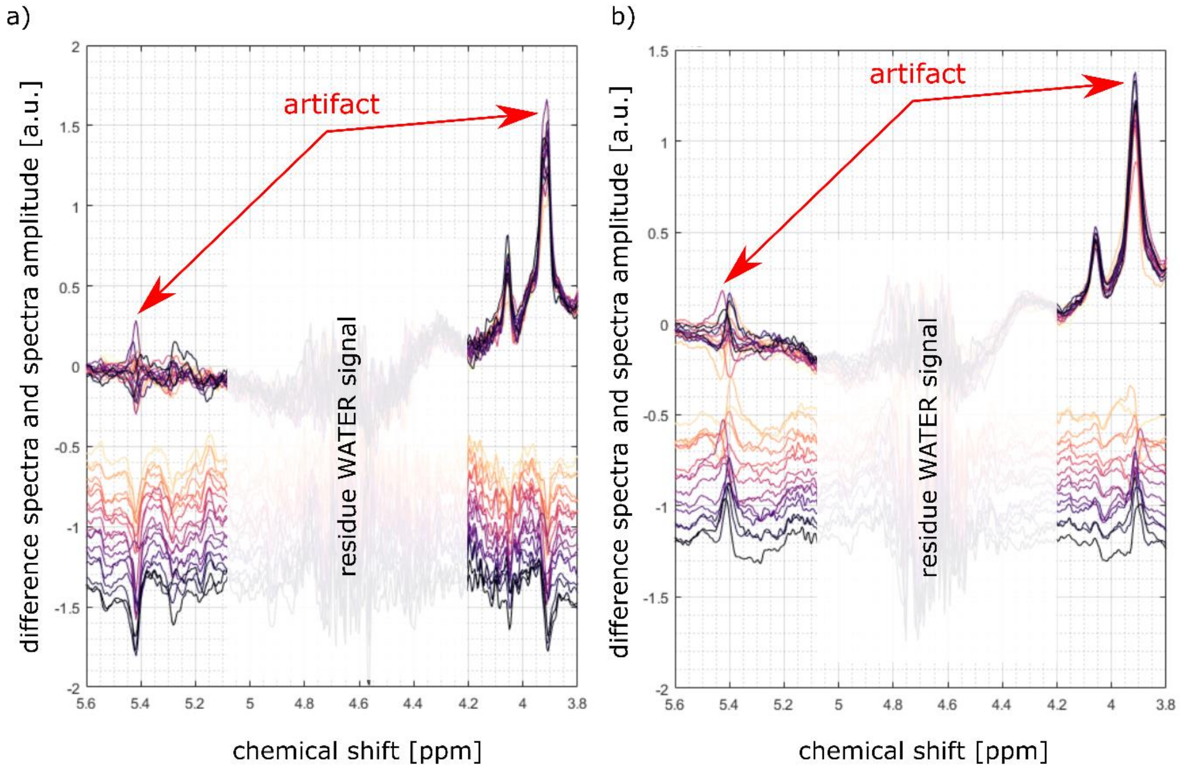

Figure S2: Spectral data and difference spectra from two volunteers with the artifact at ca. 3.9 and 5.4 ppm caused by phase jittering due to the scanner hardware. Colors indicate different time points.

Compartment Model

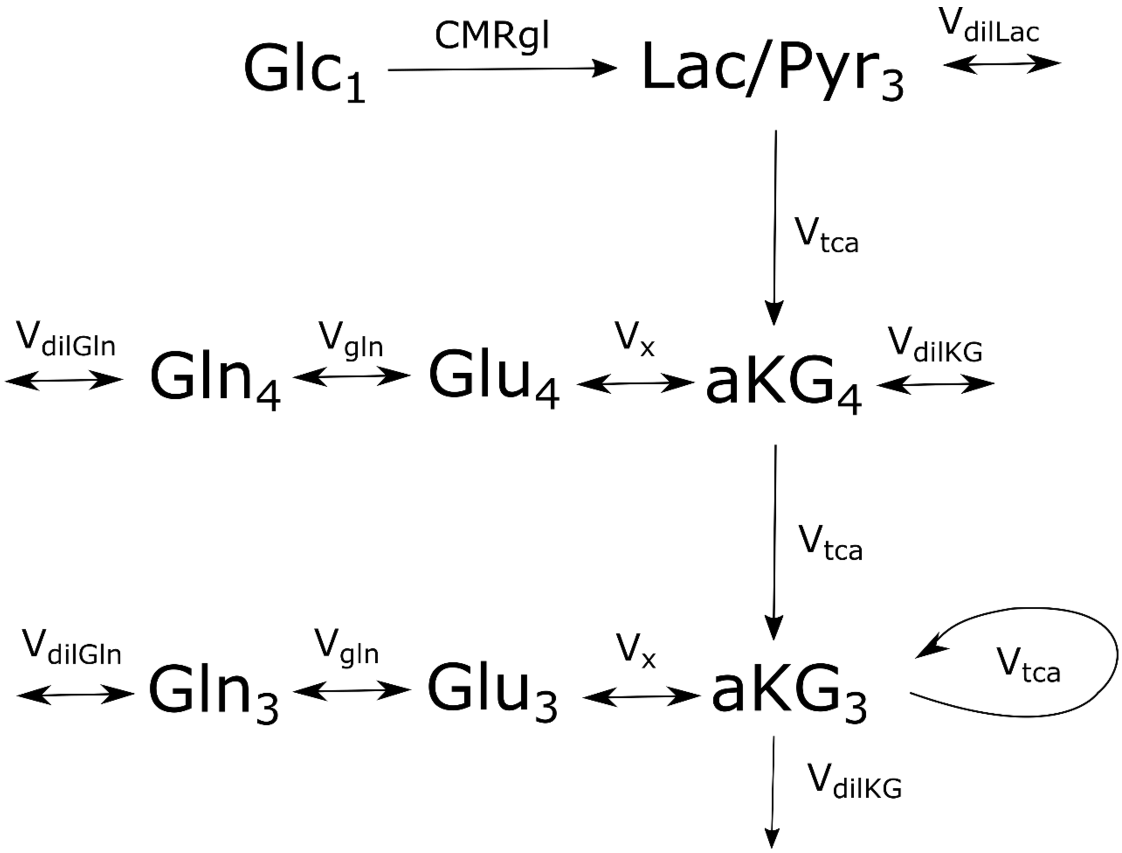

Figure S3: Simple one-compartment model to calculate the rates: the combined rate of glutamine synthetase and glutaminase Vgln, TCA cycle rate Vtca and the cytosolic amino acids/mitochondrial TCA cycle intermediates exchange rate Vx with a 1-compartment metabolic model using CWave. The rates VdilKG, VdilGln and VdilLac describe possible dilution with an unlabeled pool of the corresponding metabolite. Figures and explanations to the labeling positions were explained in previous literature, see section 2.7 for references.

Table Appendix T1: Differential equations that describe the model in Figure S3.

Mass Balance:

dL/dt = VdilL + 2CMRgl - [Vpdh + VdilL]

dGlu/dt = Vgln + Vx - [Vx + Vgln]

dGln/dt = Vgln + VdilGln - [Vgln + VdilGln]

dKG/dt = Vx + Vpdh + VdilKG - [Vx + Vtca]

dKG{1}/dt = 0.5Vtca + 0.5Vtca + Vx - [Vx + Vtca]

dGlu{1}/dt = Vgln + Vx - [Vx + Vgln]

dGln{1}/dt = Vgln + VdilGln - [Vgln + VdilGln]

Isotope Balance:

$\mathrm{d}L_3/\mathrm{d}t = \mathrm{VdilL}(\mathrm{Nat\_Abund}_0/\mathrm{Nat\_Abund}) + \mathrm{CMRgl}(\mathrm{BrainGlc}_1/\mathrm{BrainGlc}) - [\mathrm{Vpdh} + \mathrm{VdilL}](L_3/L)$

$\mathrm{dGlu}_4/\mathrm{d}t = \mathrm{Vgln}(\mathrm{Gln}_4/\mathrm{Gln}) + \mathrm{Vx}(\mathrm{KG}_4/\mathrm{KG}) - [\mathrm{Vx} + \mathrm{Vgln}](\mathrm{Glu}_4/\mathrm{Glu})$

$\mathrm{dGln}_4/\mathrm{d}t = \mathrm{Vgln}(\mathrm{Glu}_4/\mathrm{Glu}) + \mathrm{VdilGln}(\mathrm{Nat\_Abund}_0/\mathrm{Nat\_Abund}) - [\mathrm{Vgln} + \mathrm{VdilGln}](\mathrm{Gln}_4/\mathrm{Gln})$

$\mathrm{dKG}_4/\mathrm{d}t = \mathrm{Vx}(\mathrm{Glu}_4/\mathrm{Glu}) + \mathrm{Vpdh}(L_3/L) + \mathrm{VdilKG}(\mathrm{Nat\_Abund}_0/\mathrm{Nat\_Abund}) - [\mathrm{Vx} + \mathrm{Vtca}](\mathrm{KG}_4/\mathrm{KG})$

$\mathrm{dKG}\{1\}_3/\mathrm{d}t = 0.5\mathrm{Vtca}(\mathrm{KG}_4/\mathrm{KG}) + 0.5\mathrm{Vtca}(\mathrm{KG}\{1\}_3/\mathrm{KG}\{1\}) + \mathrm{Vx}(\mathrm{Glu}\{1\}_3/\mathrm{Glu}\{1\}) - [\mathrm{Vx} + \mathrm{Vtca}](\mathrm{KG}\{1\}_3/\mathrm{KG}\{1\})$

$\mathrm{dGlu}\{1\}_3/\mathrm{d}t = \mathrm{Vgln}(\mathrm{Gln}\{1\}_3/\mathrm{Gln}\{1\}) + \mathrm{Vx}(\mathrm{KG}\{1\}_3/\mathrm{KG}\{1\}) - [\mathrm{Vx} + \mathrm{Vgln}](\mathrm{Glu}\{1\}_3/\mathrm{Glu}\{1\})$

$\mathrm{dGln}\{1\}_3/\mathrm{d}t = \mathrm{Vgln}(\mathrm{Glu}\{1\}_3/\mathrm{Glu}\{1\}) + \mathrm{VdilGln}(\mathrm{Nat\_Abund}_0/\mathrm{Nat\_Abund}) - [\mathrm{Vgln} + \mathrm{VdilGln}](\mathrm{Gln}\{1\}_3/\mathrm{Gln}\{1\})$

Assumptions:

CMRgl = 0.5*Vtca

Vtca = Vpdh

FWHM and SNR of the different voxel positions

In the first row of Figure S4, the FWHM of the NAA $^{2}CH_3$ (a) and Cr CH_3 peak (b) for the occipital lobe (red) and the frontal cortex (blue) areseen for all measurements. While the median FWHM is lower for both metabolite peaks for the frontal cortex, the FWHM for the frontal cortex reveals a higher variability (the 25%- and the 75% percentile cover a larger FWHM range than the FWHM of the occipital lobe). The same is true for the FWHM of the water reference signal (e). To be more precise: For three measurements in the frontal cortex, the FWHM of water changed more than 13% while for all other volunteers, the change is less than 7% as it is the case in the occipital lobe for all volunteers.

The SNR (seen in the second row of Figure S4) is calculated as the absolute maximum of either the NAA $^{2}CH_3$ (c) or the Cr CH_3 peak (d) divided by standard deviation of the noise (in the spectral range between -1 and -4 ppm). The SNR for NAA and Cr is higher in the occipital lobe than in the frontal cortex. This can be explained by the following: The high receive SNR surface coil specifically made for SVS in the occipital lobe leads to higher SNR in the occipital lobe. In addition, the larger distance to the receive elements and the larger volume excited by the volume coil in the frontal cortex induce more noise.

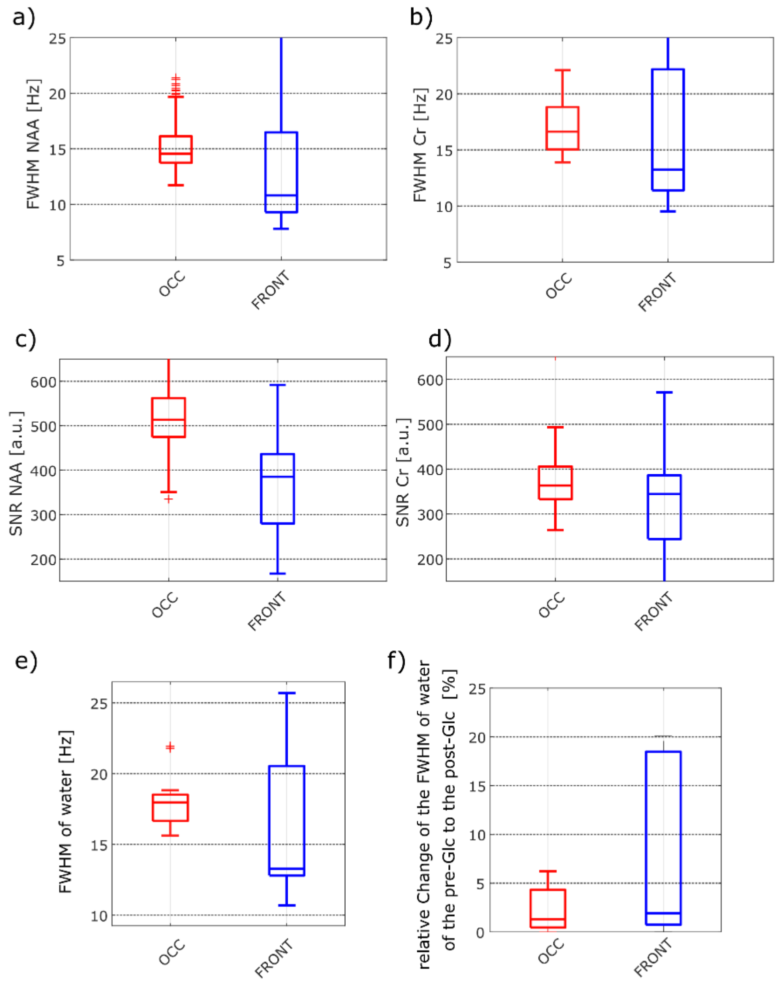

Figure S4: In the first row, the FWHM for NAA a) and Cr b) is seen for the occipital lobe (red) and the frontal cortex (blue). In the second row the SNR value of NAA c) and Cr d) is shown. In the last row the FWHM of water is seen in e) and the relative change of the FWHM of water from the pre-Glc acquisiton to the post-Glc acquisiton is presented in f)

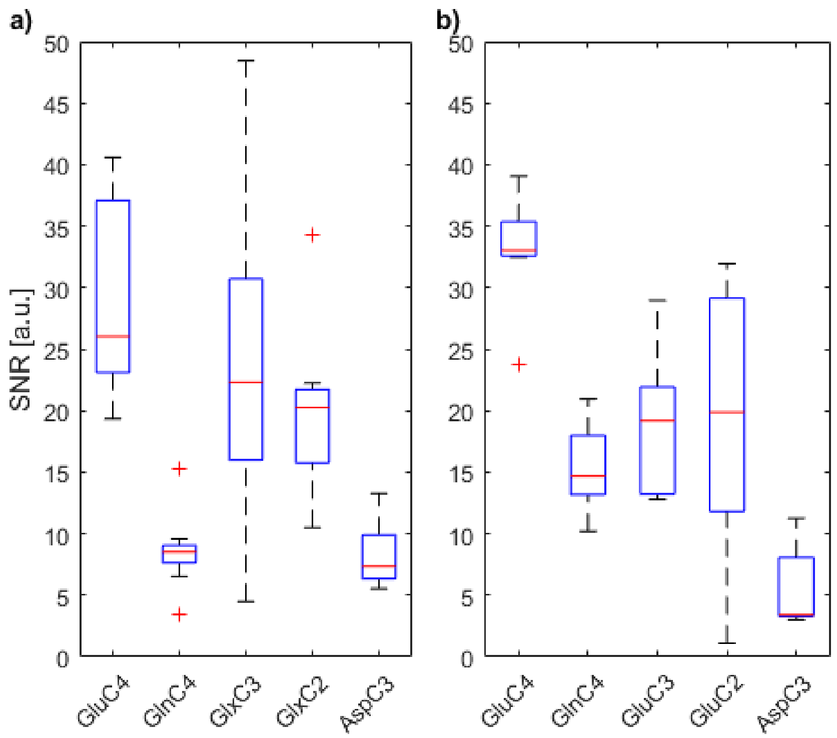

Figure S5: SNR of the [4-12C]Glu, [4-12C]Gln, [3-12C]Glx, [2-12C]Glx and [3-12C]Asp peaks from the last difference spectrum of all volunteers from the a) occipital lobe and b) frontal cortex. The SNR of the Cr CH_3 peak is about 10 times higher.

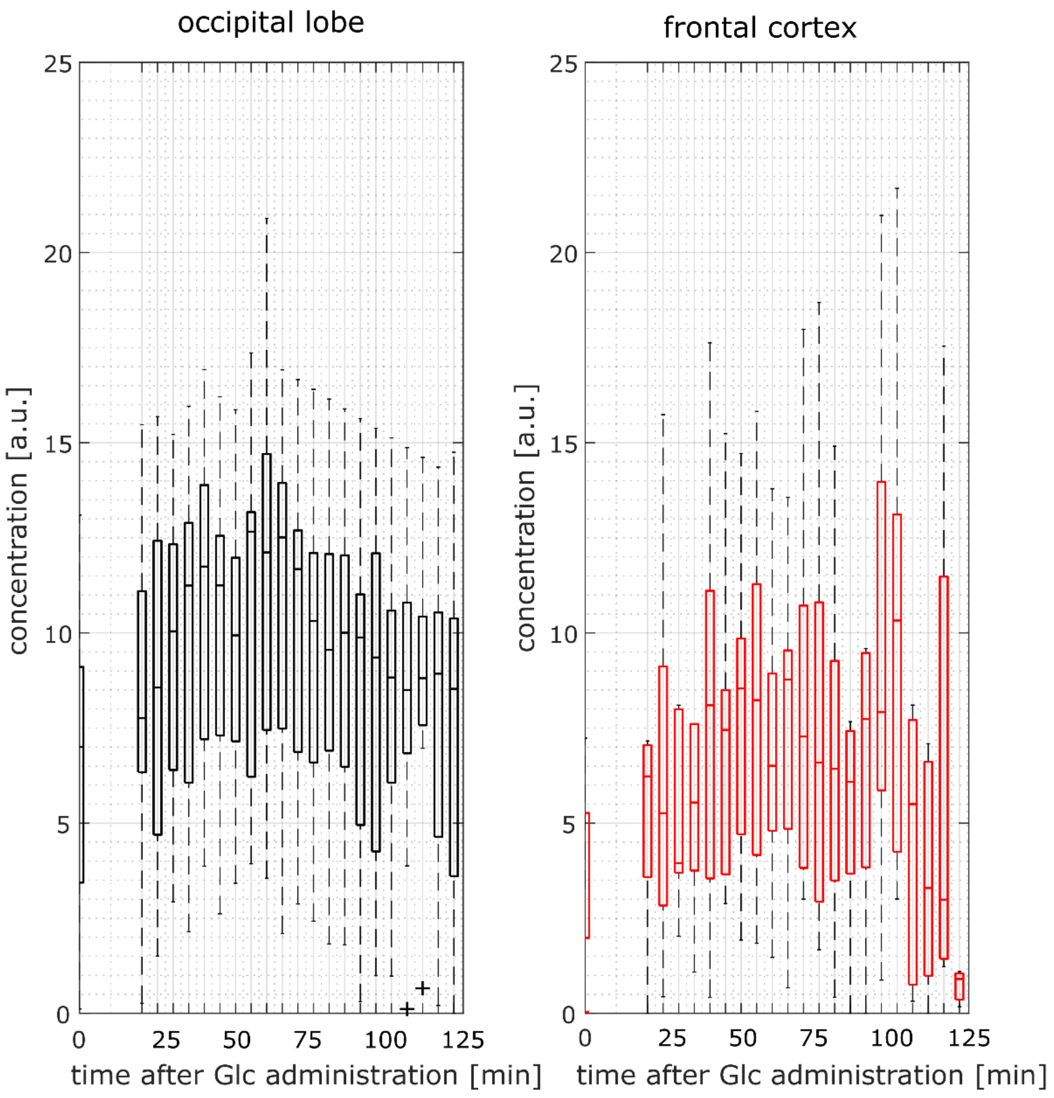

Figure S6: Boxplots of Glc total time courses for a) occipital lobe and b) frontal cortex.

1. Pfrommer A, Avdievich NI, Henning A. Four Channel Transceiver Array for Functional Magnetic Resonance Spectroscopy in the Human Visual Cortex at 9.4 T 1. In: *ISMRM*. ; 2014.

2. Dorst J, Borbath T, Landheer K, Avdievich N, Henning A. Simultaneous detection of metabolite concentration changes, water BOLD signal and pH changes during visual stimulation in the human brain at 9.4T. *J Cereb Blood Flow Metab*. January 2022:0271678X2210758. doi:10.1177/0271678X221075892

3. Avdievich NI, Giapitzakis IA, Pfrommer A, Shajan G, Scheffler K, Henning A. Decoupling of a double-row 16-element tight-fit transceiver phased array for human whole-brain imaging at 9.4 T. *NMR Biomed*. 2018;31(9):e3964. doi:10.1002/nbm.3964

4. Giapitzakis I-A, Avdievich N, Henning A. Characterization of macromolecular baseline of human brain using metabolite cycled semi-LASER at 9.4T. *Magn Reson Med*. 2018;80(2):462-473. doi:10.1002/mrm.27070

5. Blüml S, Moreno A, Hwang J-H, Ross BD. 1-13C Glucose Magnetic Resonance Spectroscopy of Pediatric and Adult Brain Disorders. *NMR Biomed*. 2001;14(1):19-32. doi:10.1002/nbm.679

6. Mason GF, Petersen KF, de Graaf RA, Kanamatsu T, Otsuki T, Rothman DL. A comparison of 13 C NMR measurements of the rates of glutamine synthesis and the tricarboxylic acid cycle during oral and intravenous administration of. *Brain Res Protoc*. 2003;10:181-190. http://www.sciencedirect.com/science/article/pii/S1385299X02002179.

7. Beckmann N, Fried R, Turkalj I, Seeling J, Keller U, Stalder G. Noninvaive observation of hepatic glycogen formation in man by13C MRS after Orl and intravenous glucose administration. *Magn Reson Med*. 1993;29(5). doi:10.1002/mrm.1910290502

8. Moreno A, Blüml S, Hwang J-H, Ross BD. Alternative 1-13C glucose infusion protocols for clinical 13C MRS examinations of the brain. *Magn Reson Med*. 2001;46(1):39-48.

doi:10.1002/mrm.1158

9. Moreno A, Ross BD, Blüml S. Direct determination of the N-acetyl-L-aspartate synthesis rate in the human brain by 13C MRS and [1-13C]glucose infusion. *J Neurochem*. 2001;77(1):347-350. doi:10.1046/j.1471-4159.2001.00282.x
